# A total blood volume or more transfused during pregnancy or after childbirth: Individual patient data from six international population-based observational studies

**DOI:** 10.1371/journal.pone.0244933

**Published:** 2021-01-22

**Authors:** Stephen J. McCall, Dacia Henriquez, Hellen McKinnon Edwards, Thomas van den Akker, Kitty W. M. Bloemenkamp, Johanna van der Bom, Marie-Pierre Bonnet, Catherine Deneux-Tharaux, Serena Donati, Ada Gillissen, Jennifer J. Kurinczuk, Zhuoyang Li, Alice Maraschini, Aurélien Seco, Elizabeth Sullivan, Simon Stanworth, Marian Knight

**Affiliations:** 1 National Perinatal Epidemiology Unit, Nuffield Department of Population Health, University of Oxford, Headington, Oxford, United Kingdom; 2 Center for Research on Population and Health, Faculty of Health Sciences, American University of Beirut, Riad El Solh, Beirut, Lebanon; 3 Department of Obstetrics and Gynaecology, Leiden University Medical Centre, Leiden, Netherlands; 4 Jon J van Rood Center for Clinical Transfusion Research, Sanquin Research & Department of Clinical Epidemiology, Leiden University Medical Center, Leiden, Netherlands; 5 Department of Obstetrics and Gynaecology, Copenhagen University Hospital Herlev, Herlev, Denmark; 6 Department of Obstetrics, Birth Centre Wilhelmina’s Children Hospital, Division Woman and Baby, University Medical Centre Utrecht, Utrecht, Netherlands; 7 Department of Anaesthesiology and Critical Care, Armand Trousseau Hospital, Assistance Publique des Hôpitaux de Paris, Paris, France; 8 INSERM U1153, Obstetrical, Perinatal and Pediatric Epidemiology Research Team (EPOPé), Research Center for Epidemiology and Biostatistics (CRESS), Paris University, Paris, France; 9 National Centre for Disease Prevention and Health Promotion, Istituto Superiore di Sanità - Italian National Institute of Health, Rome, Italy; 10 Faculty of Health and Medicine, The University of Newcastle, Newcastle, Australia; 11 Clinical Research Unit of Paris Descartes Necker Cochin, APHP, Paris, France; 12 Oxford University Hospitals NHS Trust, Department of Haematology, Oxford, United Kingdom; 13 NIHR BRC Blood Theme, University of Oxford, Oxford, United Kingdom; 14 NHS Blood and Transplant, John Radcliffe Hospital, Oxford, United Kingdom; University of Mississippi Medical Center, UNITED STATES

## Abstract

**Background:**

This study aimed to compare incidence, management and outcomes of women transfused their blood volume or more within 24 hours during pregnancy or following childbirth.

**Methods:**

Combined analysis of individual patient data, prospectively collected in six international population-based studies (France, United Kingdom, Italy, Australia, the Netherlands and Denmark). Massive transfusion in major obstetric haemorrhage was defined as transfusion of eight or more units of red blood cells within 24 hours in a pregnant or postpartum woman. Causes, management and outcomes of women with massive transfusion were compared across countries using descriptive statistics.

**Findings:**

The incidence of massive transfusion was approximately 21 women per 100,000 maternities for the United Kingdom, Australia and Italy; by contrast Denmark, the Netherlands and France had incidences of 82, 66 and 69 per 100,000 maternities, respectively. There was large variation in obstetric and haematological management across countries. Fibrinogen products were used in 86% of women in Australia, while the Netherlands and Italy reported lower use at 35–37% of women. Tranexamic acid was used in 75% of women in the Netherlands, but in less than half of women in the UK, Australia and Italy. In all countries, women received large quantities of colloid/crystalloid fluids during resuscitation (>3·5 litres). There was large variation in the use of compression sutures, embolisation and hysterectomy across countries. There was no difference in maternal mortality; however, variable proportions of women had cardiac arrests, renal failure and thrombotic events from 0–16%.

**Interpretation:**

There was considerable variation in the incidence of massive transfusion associated with major obstetric haemorrhage across six high-income countries. There were also large disparities in both transfusion and obstetric management between these countries. There is a requirement for detailed evaluation of evidence underlying current guidance. Furthermore, cross-country comparison may empower countries to reference their clinical care against that of other countries.

## Introduction

The most common form of major obstetric haemorrhage (MOH), postpartum haemorrhage (PPH) remains a major cause of maternal mortality and morbidity. PPH occurs in 3–7% of deliveries in high-income settings [1]. In France, Italy and the United States, haemorrhage is the leading cause of maternal mortality responsible for 11%, 15% and 14% of maternal deaths, respectively [2–4] and haemorrhage related mortality in the United Kingdom (UK) nearly doubled during the period 2010–12 to 2013–15 [5]. In addition, major obstetric haemorrhage (MOH) is a common reason for admission to intensive care [6–9].

Management of MOH, often defined pragmatically as transfusion of a total body blood volume (8–10 units of red blood cells) or more within 24 hours of delivery [10–12] and also referred to as massive transfusion, focusses on transfusion and fluid resuscitation, alongside planning for definitive obstetric and surgical interventions [13]. The literature in major haemorrhage caused by trauma has emphasised the importance of damage control resuscitation, including timely transfusion support, early use of coagulation factors and minimising use of crystalloids, which have all contributed to improved clinical outcomes. However, it is unclear how far these protocols should be applied in an obstetric setting [14].

Multiple guidelines to direct transfusion practice in MOH exist and these vary in terms of when and what quantity of blood components should be given. The reasons for variation in practice are unclear but are likely in part to reflect lack of high-quality data [15, 16]. A number of national studies have reported the transfusion management of PPH [13, 17, 18], but there has been little comparison reported at a national level to explore regional variations in practice. As part of an initiative to inform the international research agenda, this exploratory study was planned to describe and compare the incidence, characteristics, aetiology, management and outcomes of pregnant or postpartum women who have been transfused their total blood volume or more within 24 hours, based on regional or national data collection systems across six countries.

## Methods

### Study description

This international population-based study of massive transfusion in MOH, used secondary analyses of data from six population-based studies from Australia, Denmark, France, Italy, the Netherlands and the UK.

### Overall description

Member countries of the International Network of Obstetric Survey Systems were invited to provide national/sub national data on obstetric haemorrhage, and this included published and non-published data. In each country, data were collected either nationally or from a number of regions on a population basis using tailored data collection systems. A detailed explanation of the methodology for each country is summarised in Table 1. In short, these population-based studies collected data, using enhanced systems, from the medical records of the women who met the case definition for each of the respective studies. Danish data were solely based on information entered into the Danish Medical Birth Registry and the Danish Transfusion Database, which included data from all hospitals in Denmark. Three of the included datasets were national except for the Netherlands, France, and Italy. The TeMpOH-1 study in the Netherlands collected data from 75% of national births. Italy collected data from six regions and these represented 49% of births in Italy. The EPIMOMS study in France collected data from six regions and these regions represented 18% of national births.

**Table 1:** Summary of data collection systems and data collected.

Country	Name of system or dataset	Type of system	Date collected
Australia	Australasian Maternity Outcomes Surveillance System (AMOSS). [35]	National obstetric surveillance system. Collected data from hospitals with more than 50 births per year.	One year (July 2014-June 2015)
Denmark	Danish Medical Birth Registry and the Danish Transfusion Database	Routinely collected national data on both birth and transfusions.	Five years (2010–2015)
France	Épidémiologic de la Morbidité Maternelle Sévère study (EPIMOMS) [36]	Prospective Population-based study from all maternity units in 6 regions.	One year (2012–2013)
Italy	Italian Obstetric Surveillance System (ItOSS)	Population-based study from all maternity units in 6 regions in Italy.	Two years (September 2014-August 2016)
The Netherlands	Transfusion strategies in women during Major Obstetric Haemorrhage study (TeMpOH-1) [37]	Collected data from 75% of national births	Two years (January 2011 and January 2013)
United Kingdom	United Kingdom Obstetric Surveillance System (UKOSS) [38]	Obstetric surveillance system which collected data nationally from all consultant-led hospitals.	One year (June 2013- July 2014)

### Case definition

Massive transfusion in major obstetric haemorrhage was defined as a receipt of eight or more units of red blood cells within 24 hours in a pregnant or postpartum woman of at least 20 weeks of gestation.

### Case notification and data collection

#### United Kingdom and Australia

Both countries used a national surveillance system to identify cases of massive transfusion from consultant led obstetric units nationally in the United Kingdom and from hospitals with over 50 births per year in Australia. Reporters were asked to complete a data collection form using the medical records of the women. Data were collected anonymously. The findings from the United Kingdom study have been published previously [11, 12].

During the period 2014–2015, AMOSS identified women who had 5 or more units of RBCs transfused within 24 hours at 20 weeks’ gestation or more. Between June 2013- July 2014, women who had 8 or more units of RBCs transfused within 24 hours at 20 weeks’ gestation or more were identified using the United Kingdom Obstetric Surveillance System.

#### France

This study included data from the EPIMOMS study that prospectively identified cases of severe maternal morbidity from 6 regions in France: Alsace Champagne-Ardenne, Lorraine, Auvergne, Rhone-Alpes, Ile-de-France and Normandy. Cases were identified for a year between 2012–2013 from 113 intensive care units and 118 maternity units. Selected maternal morbidities were identified by a local reporter in each hospital. Data about demographics, previous medical history, current pregnancy, causes, management and maternal and perinatal outcomes were collected from the case notes of each women.

Major obstetric haemorrhage was one of the severe maternal morbidities included in the EPIMOMS study. To be included as a MOH case, women had to be at least 22 weeks’ of gestation or more and had to meet one of the following criteria: ≥1500mls blood loss, blood transfusion ≥ 4 RBC, uterine artery embolisation, ligation or compressive uterine sutures or have an emergency peripartum hysterectomy [19].

#### Italy

The Italian Obstetric Surveillance (ItOSS) system in Italy identified women who had 4 or more units of RBCs transfused at 22 weeks’ gestation or greater. This prospective population-based study identified women during 2014–2016 from six regions in Italy, namely Piedmont and Emilia-Romagna, Tuscany, Lazio, Campania and Sicily. The clinical reporter from each facility identified and collected data on women with PPH, uterine rupture, abnormally invasive placenta and peripartum hysterectomy. Clinical and demographic data were collected from the woman’s medical records and entered into an online data collection form. The completeness of the reported cases was verified through the Hospital Discharge Database.

#### The Netherlands

TeMpOH-1 was a national retrospective cohort study conducted between January 2011 and January 2013 in the Netherlands. Women were identified using records from blood transfusion services and birth registries of participating hospitals. Women were included if they had received four units or more of RBCs or a multicomponent blood transfusion as a result of obstetric haemorrhage. Data were collected from medical records and included information about previous medical history, current pregnancy, causes of haemorrhage, haematological parameters, blood components, fluid resuscitation and surgical and medical management.

#### Denmark

The Danish data were identified and collected using the Danish National Birth Registry (DNBR) and the Danish Transfusion Database (DTD). During the period 2010–2015, women who had a birth identified in the DNBR and had transfused 8 or more units of RBC within 24 hours identified from DTD were included in study. The DNBR collects information on all births in Denmark and this includes clinical and demographic data from each birth. The DTD is a national database and contains information of the quantity and type of blood products transfused and the time of each transfusion.

### Comparability of datasets

With reference to the literature, the clinical and demographic variables required to answer the objectives were requested from the participating countries. Available desired variables were provided by each country. Using data dictionaries from each country, each variable was mapped to assess the availability and comparability between datasets. If the components of a particular variable were not comparable between the datasets, then a common definition for that variable was created. If this was not possible the variable was excluded from the analysis. A mapping exercise was completed to assess whether the coding matched; where coding differed, a harmonised coding was devised and applied. The included variables and issues with comparability can be found in S1 Table.

### Statistical analysis

Women’s characteristics, medical history, obstetric and transfusion management and maternal outcomes were compared across countries. Normality was assessed using histograms. Normally distributed continuous variables were presented as means with standard deviations and skewed continuous variables were presented as medians with interquartile ranges. The exact binomial distribution was used to estimate confidence intervals for proportions. Statistical hypothesis testing was carried out to test for statistical differences between countries and respective characteristics, management and outcomes of women undergoing massive transfusion. Descriptive analyses used the following tests where appropriate: Analysis of Variance (ANOVA), Wilcoxon rank-sum test, Kruskal-Wallis test and chi square tests or the Fisher-Freeman-Halton's test. In order to reduce the risk of a type I error a p-value <0·01 was used to indicate statistical significance. Missing data were included as a ‘missing’ category for categorical variables. If a country did not collect the specified variable this was indicated by a dash in tables. Complete case analysis was used for continuous variables.

#### Categorisation of aetiology of MOH

France had multiple causes of MOH coded, so a primary cause was devised using a hierarchical approach. The primary cause was defined in the following order: first those with abnormal placentation, second women with abruption, third women with trauma and lastly those with atony.

### Ethics approval and data sharing agreements

This study was a secondary data analysis of data collected from primary studies. All countries had the required ethics approval for their primary studies and performed research following the World Medical Association's Declaration of Helsinki [20]. The UK, Italy, TeMpOH-1 (the Netherlands), France and Australia had obtained ethics approval for the collection of anonymised information without seeking the consent of the women from the London Multi-centre Research Ethics Committee (MREC reference 04/MRE02/45), Italian National Institute of Health (Prot. PRE‐839/13, Rome 19/12/2013), Leiden University Medical Center, National Data Protection Authority in France (CNIL, authorisation no. 912210, 14/3/2012), and from the Human Research Ethics Committee, New South Wales, Australia and from Human Research Ethics Committee for individual sites across Australia, respectively. Australia obtained ethics approval for this secondary data analysis; this study was approved by the Human Research Ethics Committee, New South Wales, Australia. The TeMpOH-1 study was approved by the ethics committee of the Leiden University Medical Center and by the institutional review board of each participating hospital; the TeMpOH-1 and ItOSS steering committees approved the study protocol and the data transfer agreement. The ethics committee at the Leiden University Medical Center approved the study protocol. The French data collaborators gained approval for this analysis from the EPIMOMS steering committee at INSERM, Paris. Ethics committee approval for the secondary analysis of UK data was not required. The Danish collaborators acquired permission from the Danish Data Protection Agency (J.nr: HGH-2016-066), no permission was required from the Danish Ethics Committee according to Danish law.

## Results

Denmark reported the highest incidence of massive transfusion at 82 (95% CI: 73–92) women per 100,000 deliveries; followed by France and the Netherlands with an incidence of 69 per 100,000 deliveries (95% CI: 58–82) and 66 per 100,000 deliveries (95% CI: 57–77), respectively. The UK, Australia and Italy had a similar incidence of massive transfusion at approximately 21 women per 100,000 deliveries (Table 2). These variable rates are seen despite many similarities in baseline characteristics of women presenting with PPH, including age and BMI.

**Table 2:** Sociodemographic, previous medical history and current pregnancy information in women undergoing massive transfusion for major obstetric haemorrhage.

		UK n = 162		Australia n = 61		Italy n = 99		the Netherlands n = 179		France n = 126		Denmark n = 288		P-value
National denominator		787,105		302,666		458,995		270,101		182,309		349,998		
Incidence per 100,000 maternities		20·6 (17·5–24·0)		20·2 (15·4–25·9)		21·6(17·5–26·3)		66·3 (56·9–76·7)		69·1 (57·9–82·3)		82·3 (73·1–92·4)		
**Age**	*Mean (Std)*	33	(5·9)	33	(5·3)	35	(6·1)	32	(5·3)	33	(5·9)	32	(5·4)	<0·001
**BMI**	*Median (IQR)*	25	(22–29)	25	(21–28)	23	(21–25)	23	(21–26)	24	(21–27)	23	(21–27)	<0·001
*Previous medical history*														
**Parity**	*Nulliparous*	60	(37·3)	20	(32·8)	48	(48·5)	84	(46·9)	56	(44·8)	128	(44·8)	0·185
	*Multiparous*	101	(62·7)	41	(67·2)	51	(51·5)	95	(53·1)	69	(55·2)	158	(55·2)	
	*Missing*	1		0		0		0		1		2		
**Previous caesarean section**	*None*	35	(34·3)	25	(61)	23	(46·9)	51	(53·7)	38	(55·9)	77	(48·1)	0·020
	*Yes*	67	(65·7)	16	(39)	26	(53·1)	44	(46·3)	30	(44·1)	83	(51·9)	
	*Nulliparous*	60		20		48		84		56		128		
	*Missing*	0		0		2		0		2		0		
**Number of previous caesarean section**	*0*	35	(34·3)	25	(61)	23	(46·9)	-	-	38	(55·9)	-	-	0.036
	*1*	38	(37·3)	10	(24·4)	16	(32·7)	-	-	21	(30·9)	-	-	
	*2+*	29	(28·4)	6	(14·6)	10	(20·4)	-	-	9	(13·2)	-	-	
	*Nulliparous*	60		20		48		-	-	56		-	-	
	*Missing*	0		0		2				2				
**Previous postpartum haemorrhage**	*None*	78	(76·5)	29	(85·3)	41	(91·1)	86	(91·5)	64	(92·8)	-	-	0·007
	*Yes*	24	(23·5)	5	(14·7)	4	(8·9)	8	(8·5)	5	(7·2)	-	-	
	*Nulliparous*	60		20		48		84		56		-	-	
	*Missing*	0		7		6		1		1		-	-	
*Current pregnancy*														
**Hypertension prior to pregnancy or current problem**	*None*	161	(99·4)	59	(96·7)	94	(94·9)	153	(85·5)	124	(99·2)	-	-	<0·001
	*Yes*	1	(0·6)	2	(3·3)	5	(5·1)	26	(14·5)	1	(0·8)	-	-	
	*Missing*	0		0		0		0		1		-	-	
**Infection in current pregnancy**	*No*	161	(99·4)	56	(94·9)	99	(100)	179	(100)	117	(97·5)	-	-	0·006*
	*Yes*	1	(0·6)	3	(5·1)	0	(0)	0	(0)	3	(2·5)	-	-	
	*Missing*	0		2		0		0		6		-	-	
**Inherited bleeding disorders**	*No*	160	(98·8)	55	(98·2)	-	-	-	-	121	(96·8)	-	-	0·602*
	*Yes*	2	(1·2)	1	(1·8)	-	-	-	-	4	(3·2)	-	-	
	*Missing*	0		5		-	-	-	-	1		-	-	
**Multiple pregnancy**	*No*	155	(95·7)	58	(95·1)	92	(92·9)	167	(93·8)	108	(85·7)	219	(76)	<0·001
	*Yes*	7	(4·3)	3	(4·9)	7	(7·1)	11	(6·2)	18	(14·3)	69	(24)	
	*Missing*	0		0		0		1		0		0		
**Induced**	*None*	103	(65·2)	20	(54·1)	56	(73·7)	117	(65·4)	52	(41·6)	-	-	<0·001
	*Yes*	55	(34·8)	17	(45·9)	20	(26·3)	62	(34·6)	73	(58·4)	-	-	
	*Missing*	4		24		23		0		1		-	-	
**Delivery type**	*Vaginal delivery*	50	(31·6)	25	(41)	37	(37·4)	104	(58·4)	47	(37·6)	92	(31·9)	<0·001
	*Caesarean delivery*	108	(68·4)	36	(59)	62	(62·6)	74	(41·6)	78	(62·4)	196	(68·1)	
	*N/A Pregnancy loss*	4		0		0		1		1		0		

*Used Fisher’s exact test.

Missing data were not included in the percentage. A dash indicated that the country did not collect data on that specific variable.

Mean age of women with massive transfusion was 32 years (SD ±5·7), and the majority were multiparous (57%). Amongst women with a previous pregnancy, there were differences between countries in the proportion of women with previous caesarean section and previous postpartum haemorrhage (previous caesarean section: highest in the UK (66%) and lowest in Australia (39%)).

Amongst cases, there were differences in the proportions of women with multiple pregnancies between countries (Denmark 24%, France 14% and UK 4%, respectively). In addition, the majority of women were delivered by caesarean section in each country (range excluding the Netherlands: 59%-68%), while the Netherlands had the lowest proportion of caesarean deliveries at 42%.

The most common cause of MOH in all countries was atony, with a prevalence ranging from 40% in the UK to 63% in the Netherlands (S2 Table). The second leading cause was abnormal placentation with a prevalence ranging from 22% in Italy and the Netherlands to 32% in Australia. The least common cause was placental abruption with a proportion ranging from 3% in the Netherlands and Italy to 9% in the UK.

Use of blood components varied across countries, Italy had a smaller median use of fresh frozen plasma (FFP) and platelets compared to other countries (Table 3). France had the highest use of FFP with a median of 7 units (IQR: 5–9) compared with the other countries. The inter country difference in use of FFP was statistically significant (P<0·01).

**Table 3:** Haematological management by country.

Blood products		UK n = 162		Australia n = 61		Italy n = 99		the Netherlands n = 179		France n = 126		Denmark n = 288		P-value
**Number of RBC units**	*Median (IQR)*	10	(8–14)	11	(9–14)	9	(8–11)	11	(9–15)	10	(9–12)	11	(8–15)	<0·001
**Received Fresh frozen plasma**	*n (%)*	162	(100)	59	(96·7)	84	(84·8)	179	(100)	126	(100)	247	(85·8)	<0·001
**Fresh frozen plasma**	*Median (IQR)*	6	(4–8)	6	(4–8)	4	(2–8)	6	(4–8)	7	(5–9)	6	(4–8)	<0·001
**Received Platelets**	*n (%)*	126	(77·8)	51	(83·6)	40	(40·4)	179	(100)	126	(100)	205	(71·2)	<0·001
**Platelets**	*Median (IQR)*	1	(1–2)	1	(1–2)	0	(0–2)	2	(1–3)	1	(1–3)	2	(0–3)	<0·001
**Source of concentrated fibrinogen administered**	*No*	58	(35·8)	8	(13·6)	62	(62·6)	116	(64·8)	27	(22·3)	-	-	<0·001
	*Yes*	104	(64·2)	51	(86·4)	37	(37·4)	63	(35·2)	94	(77·7)	-	-	
	*Missing*	0		2		0		0		5		-	-	
Fibrinogen concentrate used	*No*	152	(93·8)	54	(100)	62	(62·6)	116	(64·8)	27	(22·3)	-	-	<0·001
	*Yes*	10	(6·2)	0	(0)	37	(37·4)	63	(35·2)	94	(77·7)	-	-	
	*Missing*	0		7		0		0		5		-	-	
Cryoprecipitate used	*No*	55	(35·5)	8	(13·6)	-	-	-	-	-	-	-	-	0·002
	*Yes*	100	(64·5)	51	(86·4)	-	-	-	-	-	-	-	-	
	*Missing*	7		2		-	-	-	-	-	-	-	-	
Number of units of cryoprecipitate	*Median (IQR)*	2	(0–2)	1	(1–2)	-	-	-	-	-	-	-	-	0.07
**Use of cell salvage**	*No*	115	(74·2)	46	(85·2)	-	-	-	-	-	-	-	-	0·098
	*Yes*	40	(25·8)	8	(14·8)	-	-	-	-	-	-	-	-	
	*Missing*	7		7		-	-	-	-	-	-	-	-	
**If Yes, amount transfused (ml)**	*Median (IQR)*	1150	(500–1900)	884	(565–1377)	-	-	-	-	-	-	-	-	0.95
**Recombinant Factor VIIa**	*No*	149	(92)	48	(90·6)	86	(86·9)	151	(84·4)	103	(85·1)	-	-	0·224
	*Yes*	13	(8)	5	(9·4)	13	(13·1)	28	(15·6)	18	(14·9)	-	-	
	*Missing*	0		8		0		0		5		-	-	
**Tranexamic acid**	*No*	87	(53·7)	41	(74·5)	57	(57·6)	45	(25·1)	47	(39·2)	-	-	<0·001
	*Yes*	75	(46·3)	14	(25·5)	42	(42·4)	134	(74·9)	73	(60·8)	-	-	
	*Missing*	0		6		0		0		6		-	-	
Any fluid used during resuscitation**	*No*	4	(2·5)	42	(77·8)	0	(0)	0	(0)	0	(0)	-	-	<0·001*
	*Yes*	157	(97·5)	12	(22·2)	55	(100)	167	(100)	108	(100)	-	-	
	*Missing*	1		7		44		12		18		-	-	
**Total amount (ml)**		4000	(3000–6000)			3500	(3000–5000)	4500	(3000–6000)					0·205
Colloid	*Yes*	139	(88·5)	-	-	49	(98)	160	(96·4)	87	(82·9)	-	-	<0·001*
	*Missing*	5		-	-	49		13		21		-	-	
If Yes, amount transfused (ml)	*Median (IQR)*	1000	(650–2000)	-	-	1000	(500–1500)	1500	(1000–2000)	-	-	-	-	<0·001
Crystalloid	*Yes*	152	(95)	-	-	49	(100)	137	(100)	103	(95·4)	-	-	0·013*
	*Missing*	2		-	-	50		42		18		-	-	
If Yes, amount transfused (ml)	*Median (IQR)*	3000	(1500–4000)	-	-	2500	(1700–3500)	2500	(1600–4300)	-	-	-	-	0·442

*Used Fisher’s exact test.

**Australia only recorded use of 5 and 25% albumin administration.

Missing data were not included in the percentage. A dash indicated that the country did not collect data on that specific variable.

Different concentrated sources of fibrinogen were used, the UK and Australia mainly administered cryoprecipitate while France, Italy and the Netherlands used fibrinogen concentrate. The highest proportion of women receiving a fibrinogen product was in Australia (86%), while the Netherlands and Italy had the lowest use (35–37%) compared to the other countries (64%-86%).

Between 13–16% of women received factor VIIa in Italy, France and the Netherlands while the United Kingdom reported the lowest use at 8%, although this difference was not statistically significant. Use of tranexamic acid (TXA) also varied between countries, the Netherlands used TXA in 75% of women with massive transfusion while it was used in less than a half of women in the UK, Australia and Italy, and these differences were statistically significant. Large amounts of colloid and crystalloid fluids were transfused; the median fluid transfusion was between 3.5 and 4.5 litres in Italy, the Netherlands and the UK.

The use of surgical techniques and interventional radiology also varied between countries (Table 4). An intrauterine balloon was used in at least half of all women with massive transfusion except in France where a balloon was only used in a third of women. Use of arterial embolisation and major vessel ligation was more common in France and the Netherlands (71% and 52% of women, respectively) compared to the UK and Italy (16% and 4% of women, respectively).

**Table 4:** Obstetric management by country.

		UK n = 162		Australia n = 61		Italy n = 99		the Netherlands n = 179		France n = 126		Denmark n = 288		P-value
**Medical management**		** **	** **	** **	** **	** **	** **	** **	** **	** **	** **	** **	** **	** **
**Oxytocin**	*No*	6	(3·7)	7	(11·7)	26	(26·3)	65	(36·3)	47	(39·5)	-	-	<0·001
	*Yes*	156	(96·3)	53	(88·3)	73	(73·7)	114	(63·7)	72	(60·5)	-	-	
	*Missing*			1		0				7		-	-	
**Ergometrine**	*No*	69	(42·6)	32	(56·1)	-	-	160	(89·4)	-	-	-	-	<0·001
	*Yes*	93	(57·4)	25	(43·9)	-	-	19	(10·6)	-	-	-	-	
	*Missing*			4								-	-	
**Prostaglandin**	*No*	64	(39·5)	30	(52·6)	49	(49·5)	30	(16·8)	16	(13·6)	-	-	<0·001
	*Yes*	98	(60·5)	27	(47·4)	50	(50·5)	149	(83·2)	102	(86·4)	-	-	
	*Missing*			4		0				8		-	-	
**Misoprostol**	*No*	73	(45·1)	29	(51·8)	-	-	117	(65·4)	118	(99·2)	-	-	<0·001
	*Yes*	89	(54·9)	27	(48·2)	-	-	62	(34·6)	1	(0·8)	-	-	
	*Missing*			5						7		-	-	
**Any uterotonic treatment**	*No*	5	(3·1)	3	(5)	21	(21·2)	13	(7·3)	9	(7·5)	-	-	<0·001
	*Yes*	157	(96·9)	57	(95)	78	(78·8)	166	(92·7)	111	(92·5)	-	-	
	* *			1						6		-	-	
**Surgical management**	* *													
**Intrauterine balloons**	*No*	68	(42)	26	(44·8)	45	(45·5)	77	(43)	88	(72·1)	246	(85·4)	<0·001
	*Yes*	94	(58)	32	(55·2)	54	(54·5)	102	(57)	34	(27·9)	42	(14·6)	
	*Missing*	0		3		0		0		4				
Intra-abdominal packing*	*No*	147	(90·7)	47	(83·9)	-	-	-	-	109	(90·1)	-	-	0·344
	*Yes*	15	(9·3)	9	(16·1)	-	-	-	-	12	(9·9)	-	-	
	*Missing*			5		-	-	-	-	5		-	-	
**Embolisaton or ligation**	*No*	136	(84)	41	(67·2)	95	(96)	86	(48)	36	(29·5)	286	(99·3)	<0·001
	*Yes*	26	(16)	20	(32·8)	4	(4)	93	(52)	86	(70·5)	2	(0·7)	
	*Missing*									4				
**Compressive sutures**	*No*	123	(75·9)	38	(64·4)	86	(86·9)	154	(86)	90	(73·8)	247	(85·8)	<0·001
	*Yes*	39	(24·1)	21	(35·6)	13	(13·1)	25	(14)	32	(26·2)	41	(14·2)	
	*Missing*	0		2		0		0		4				
**Hysterectomy**	*No*	87	(53·7)	29	(47·5)	26	(26·3)	126	(70·4)	66	(54·5)	223	(77·4)	<0·001
	*Yes*	75	(46·3)	32	(52·5)	73	(73·7)	53	(29·6)	55	(45·5)	65	(22·6)	
** **	*Missing*	0		0		0		0		5		0		

* Italian and Danish data include intrauterine packing.

The Netherlands: intra-abdominal packing was after hysterectomy and was not included. Missing data were not included in the percentage. A dash indicated that the country did not collect data on that specific variable.

Uterine compression sutures were more commonly used in Australia, France and the UK (36%-24%) compared to the remaining countries (13–14%). In Italy, 74% of women with a massive transfusion had a hysterectomy, compared with less than half of women in the United Kingdom, France, and a third of women in the Netherlands.

Overall there were fourteen maternal deaths which gave a case fatality of 1.5% (95% CI: 0.8–2.6) (Table 5). Nevertheless, maternal mortality was rare across all countries. Fifteen women had a thrombotic event; women in France had about double the proportion of these events compared to the UK and Australia (6% vs. ~3%, respectively). In addition, France had a higher proportion of renal failure compared to other countries (16% vs. ~1–2%, respectively). Furthermore, France had a slightly higher proportion of cardiac arrests compared to other countries (8% vs. ~1–5%).

**Table 5:** Maternal outcomes by country.

		UK n = 162		Australia n = 61		Italy n = 99		the Netherlands n = 179		France n = 126		Denmark n = 288		P-value
**Maternal outcome**		** **	** **	** **	** **	** **	** **	** **	** **	** **	** **	** **	** **	** **
**Maternal death**	*No*	160	(98·8)	61	(100)	96	(97)	175	(97·8)	123	(97·6)	286	(99·3)	0·37*
	*Yes*	2	(1·2)	0	(0)	3	(3)	4	(2·2)	3	(2·4)	2	(0·7)	
**Cardiac arrest**	*No*	154	(95·1)	57	(96·6)	94	(94·9)	-	-	116	(92·1)	285	(99)	0·005*
	*Yes*	8	(4·9)	2	(3·4)	5	(5·1)	-	-	10	(7·9)	3	(1)	
	*Missing*	0		2		0				0		0		
**Renal failure**														
	*No*	160	(98·8)	58	(98·3)	97	(98)	-	-	106	(84·1)	284	(98·6)	<0·001*
	*Yes*	2	(1·2)	1	(1·7)	2	(2)	-	-	20	(15·9)	4	(1·4)	
	*Missing*			2						0		0		
**Infection**														
	*No*	157	(96·9)	56	(96·6)	97	(98)	-	-	118	(93·7)	-	-	0·399*
	*Yes*	5	(3·1)	2	(3·4)	2	(2)	-	-	8	(6·3)	-	-	
	*Missing*	0		3		0				0				
**Thrombotic event**	*No*	158	(97·5)	57	(96·6)	99	(100)	-	-	118	(93·7)	287	(99·7)	0·001*
	*Yes*	4	(2·5)	2	(3·4)	0	(0)	-	-	8	(6·3)	1	(0·3)	
	*Missing*	0		2		0		-	-	0		0		
**ITU admission**	*No*	30	(18·5)	2	(3·3)	15	(15·3)	32	(17·9)	44	(34·9)	-	-	<0·001
** **	*Yes*	132	(81·5)	59	(96·7)	83	(84·7)	147	(82·1)	82	(65·1)	-	-	

*Used Fisher’s exact test.

Maternal outcomes: Cardiac arrest: UK included—cardiac arrest, cardiac infection and cardioversion and inotropic support; Italy, Australia and France: cardiac arrest. Infection: UK: chest infection, septicaemia, septic shock, sepsis, enterococcus infection, necrotising fasciitis, C. difficile, abscess, suspected tuberculosis or meningitis, wound infection and wound dehiscence. Australia: sepsis. Italy and France: septicaemia. Thrombotic: pulmonary embolism or deep venous thrombosis.

Missing data were not included in the percentage. A dash indicated that the country did not collect data on that specific variable. ITU: Intensive therapy unit.

## Discussion

This population-based study has shown a five-fold variation in the incidence of massive transfusion across six high-income countries with similarly well-developed healthcare systems and medical resources. Despite the same primary evidence being available to clinicians and guideline writers, in each country different obstetric and haematological management is provided for the management of women undergoing transfusion in major obstetric haemorrhage. Although there were no differences in maternal mortality between countries, these numbers are low, and there was evidence of highly variable rates of cardiac arrest, thrombotic events and renal failure. The greatest variation in resuscitation was seen for use of fibrinogen products, tranexamic acid and recombinant factor VIIa. Again, there was striking variation in obstetric management including the use of intrauterine balloons, embolisation and hysterectomy between countries.

The variation in incidence between countries is likely to be multifactorial. Variations in obstetric management may be a relevant factor, for example, differential thresholds for peripartum hysterectomy may be a potential determinant of the inter country differences. More timely recognition, and control of bleeding may prevent worsening to more severe bleeds (≥8 units), and therefore factors that delay presentation and definitive management need to be explored between countries. National support for robust MOH protocols through appropriate fluid and haematological management may also control the bleeding at earlier stages. Other factors include case-mix and variation in risk factors such as the caesarean section rate, and rate of multiple pregnancies. However, the data collected in each country did not include information about the general obstetric population, so there may be other differences in background risks for PPH between countries that have not been captured. Alternatively, the variation in incidence of massive transfusion may be a reflection of differential transfusion policies.

Guidelines for the haematological and fluid resuscitation management of haemorrhage vary by country and medical specialty [16]. Consequently, it is not surprising that clinical practice and use of blood components differed by country.

Clinicians in the UK and Australia mainly used cryoprecipitate as a source of concentrated fibrinogen while in Italy, France and the Netherlands clinicians only used fibrinogen concentrate. There is a paucity of evidence regarding the efficacy of, optimal source and dose of fibrinogen. The FIB-PPH trial compared the use of fibrinogen concentrate to placebo in the initial treatment of PPH, but the intervention did not reduce the primary outcome of postpartum bleeding [21]; the study explored the efficacy of fibrinogen concentrate among a wide range of cases including non-severe PPH. The FIBRES trial conducted in cardiac surgery showed that fibrinogen was noninferior to cryoprecipitate [22]. While this study focused on sources of concentrated fibrinogen, FFP is also a source of fibrinogen; however, FFP has a much lower concentration of fibrinogen than fibrinogen concentrate or cryoprecipitate. Consequently, FFP is not recommended as a primary agent for fibrinogen concentration [23, 24].

At the time of the data collection for this study there was a lack of high-quality evidence about the use of TXA in the treatment of obstetric haemorrhage, and there was variation in use. Trials were either too small to detect a statistically significant difference in the primary outcome [25] or did not have the power to examine safety issues of the drug [26]. The findings of the WOMAN trial have now been published and it would be of interest to understand how this trial has changed TXA use [27].

In the majority of countries, current guidelines and expert reviews recommend that recombinant factor VIIa should not be given in obstetric haemorrhage [16, 28, 29]. A Cochrane review reported no evidence to support the efficacy of recombinant factor VIIa across a range of clinical settings as prophylaxis or therapeutically; but with evidence to indicate an increased risk of thromboembolic events [30]. French guidelines allow for the use of recombinant factor VIIa as a “compassionate treatment” to avoid a hysterectomy in nulliparous women or for life-threatening situations [31]. Overall, despite lack of evidence of benefit alongside risks and high costs, approximately 12% of women across all 6 countries received the pro-haemostatic agent in this international study.

Previous research has shown that obstetric management of PPH varies internationally [32–34]. The findings from this study are consistent with this, as embolisation use was higher in France and the Netherlands in comparison to the UK. This most likely reflects the availability of the infrastructure required to provide an embolisation service as well as perceived efficacy. Intrauterine balloon tamponade is the most common second stage management of MOH and its lower use in France most likely reflects higher use of embolisation.

This study also showed a significant disparity in the use of hysterectomy where three-quarters of women had a hysterectomy in Italy while only a third of women did so in the Netherlands and less than a fifth in Denmark. Consistent with previous research, a similar proportion of women in the UK and France had a hysterectomy [32]. With no difference in maternal mortality between countries, this suggests that the future fertility of some of these women could have been saved.

There was no difference in maternal mortality between countries. However, this study may not have been adequately powered to test for differences in this rare event. A higher number of women with massive transfusion in France had renal failure, cardiac arrests and thrombotic events. The reasons are unclear but higher rates of pro-haemostatic agents were reported. However, the EPIMOMS study included specific questions on these outcomes within their data collection form, which may have resulted in better ascertainment than the UK, Italy and Australia.

This study was unique in its ability to compare all women between populations, based on national or regional studies with women who had been transfused at least eight of more units of red blood cells. This study used similar methods to an individual patient data meta-analysis. This methodology allowed the harmonisation of definitions and creation of comparable datasets, which in turn allowed robust comparisons between nations. All countries except for Denmark had tailored data collection forms, which collected detailed information about each woman, and enabled this unique comparison.

This analysis should be interpreted in the context of its limitations. Each national study collected slightly different items. To reduce errors due to misclassification all countries had their cases cross-checked against hospital records, other than Denmark, which was solely based on ICD-10-CM codes.

This analysis was also susceptible to survival bias, as those women who died or had a hysterectomy before they received 8 units of RBC would not have been included in the study population. To overcome this limitation, future epidemiological studies should use a composite of inclusion criteria to prevent survival bias. This composite should include women who died from a haemorrhage, women who have a hysterectomy or surgical management due to uncontrolled bleeding, women who meet a certain number of RBC units transfused or where a major obstetric haemorrhage protocol has been activated. Furthermore, women with haemorrhage before 20 weeks of gestation were not included in the case definition, thus most women with a major haemorrhage as a result of an ectopic pregnancy would have been excluded. In addition, the surveillance systems in UK, Australia and Italy may not have identified women with secondary PPH who were not readmitted to an obstetric unit, whilst hospital databases and blood banks in Denmark, the Netherlands and France were interrogated to ensure obstetric related haemorrhages after pregnancy were included. It is unlikely, however, that differences in registration are responsible for the marked variation seen in incidence.

## Conclusion

There was a considerable variation in the incidence of massive transfusion associated with major obstetric haemorrhage between countries. This may be the result of disparities in the timely management of less severe bleeds between countries or differences in transfusion policies; however, more research is required to ascertain reasons for these differences. Obstetric management and the use of blood products and haemostatic agents varied substantially. However, despite these variations in management, the rate of maternal mortality was similar. Therefore, there is need for a detailed evaluation of the evidence underlying current guidance, including the use of fibrinogen concentrate and other pro-thrombotic agents.

## Summary

In the absence of high-level evidence, each country has a preferred treatment regimen, which may lead to the use of unnecessary costly haematological products, exposure of women to potential adverse effects and radical management such as hysterectomy. With wide variation in obstetric practice, including hysterectomy and embolisation, significant improvement in the evidence base is required particularly if a woman’s future fertility could be otherwise preserved. In extreme scenarios there is a delicate balance between saving a woman’s life and her fertility, however, with a 40% difference in use of hysterectomy between Italy and the Netherlands and no difference in maternal mortality, there must be an examination and comparison of the clinical pathway to ascertain if hysterectomy was necessary.

## Supporting information

S1 Table Availability and comparability of variables from each respective dataset. (DOCX)

S2 Table Aetiology of the massive obstetric haemorrhage by country. Legend: France had a hierarchy of primary cause of PPH and were coded using this system: 1st Abnormal placentation, 2nd Abruption, 3rd Trauma and 4th was Atony. Denmark did not have data on cause of haemorrhage.
(DOCX)
