# Supplementary material for: A total blood volume or more transfused during pregnancy or after childbirth: Individual patient data from six international population-based observational studies
Source: PLoS One. 2021 Jan 22;16(1):e0244933. doi: 10.1371/journal.pone.0244933 (PMC7822517; doi:10.1371/journal.pone.0244933)
Supplement: S1 Table — (DOCX) [file pone.0244933.s001.docx]

S1 Table. Availability and comparability of variables from each respective dataset.

| **Desired variable** | | **United Kingdom (UKOSS)** | **Australia (AMOSS)** | **Italy (ItOSS)** | **the Netherlands (TeMpOH-1)** | **France (EPIMOMS)** | **Denmark** | **Included in analysis** | **Comment** |
| --- | --- | --- | --- | --- | --- | --- | --- | --- | --- |
|  | *Sociodemographic characteristics* | | | | | | | |  |
| Age | | *Yes* | *Yes* | *Yes* | *Yes* | *Yes* | Yes | Yes |  |
| Height | | *Yes* | *Yes* | *Yes* | *Yes* | *Yes* | Yes | Yes |  |
| Weight | | *Yes* | *Yes* | *Yes* | *Yes* | *Yes* | Yes | Yes |  |
| BMI at booking | | *Yes* | *Yes* | *Yes* | *Yes* | *Yes* | Yes | Yes |  |
| Smoking status | | *Yes* | *Yes* | *No* | *No* | *Yes* | No | No | *Not considered important for research question* |
| Ethnicity | | *Yes* | *No* | *No* | *Yes* | *No* | No | No |  |
| Country of birth | | *No* | *No* | *Yes* | *No* | *Yes* | No | No |  |
| Socioeconomic status | | *No* | *Yes* | *No* | *No* | *No* | No | No |  |
| Employment status | | *Yes* | *No* | *No* | *No* | *No* | No | No |  |
| Drug use | | *No* | *No* | *No* | *No* | *Yes* | No | No |  |
| *Previous medical history* | |  |  |  |  |  |  |  |  |
| Parity | | *Yes* | *Yes* | *Yes* | *Yes* | *Yes* | Yes | Yes |  |
| Previous caesarean section | | *Yes* | *Yes* | *Yes* | *Yes* | *Yes* | Yes | Yes |  |
| Number of previous caesareans | | *Yes* | *Yes* | *Yes* | *Yes* | *Yes* | No | Yes |  |
| Previous pregnancy problems | | *Yes* | *Yes* | *No* | *Yes* | *Yes* | No | No |  |
| Previous postpartum haemorrhage | | *Yes* | *Yes* | *Yes* | *Yes* | *Yes* | No | Yes |  |
| Inherited bleeding disorders | | *Yes* | *Yes* | *No* | *Yes* | *Yes* | No | Yes |  |
| Thrombocytopenia | | *Yes* | *Yes* | *No* | *No* | *Yes* | No | No |  |
| Pre-existing medical problems | | *Yes* | *See comment* | *No* | *Yes* | *Yes* | No | No | *Used a singular code on previous medical problems* |
| Anaemia before pregnancy/ before bleeding event | | *Yes* | Yes | *No* | *No* | *Yes* | No | No | *Not comparable between countries* |
| Known infection | | *Yes* | Yes | *No* | *Yes* | *Yes* | No | Yes |  |
| Known hypertension | | *Yes* | Yes | *No* | *Yes* | *Yes* | No | Yes |  |
| *Current pregnancy characteristics* | |  |  |  |  |  |  |  |  |
| Multiple pregnancy | | *Yes* | *Yes* | *Yes* | *Yes* | *Yes* | *Yes* | *Yes* |  |
| Induction of labour | | *Yes* | *Yes* | *Yes* | *Yes* | *Yes* |  | *Yes* |  |
| Use of uterotonics in vaginal deliveries | | *No* | *Yes* | *No* | *Yes (not complete)* | *Yes* | *No* | *No* | *Was not comparable between countries* |
| Caesarean delivery | | *Yes* | *Yes* | *Yes* | *Yes* | *Yes* | *Yes* | *Yes* |  |
| Abnormal placentation during pregnancy | | *Yes* | *Yes (from coding)* | *Yes* | *No* | *Yes* | *No* | *No* | *Was not included as it was captured in the primary cause* |
| When did the haemorrhage occur? | | *Yes* | *Yes* | *No* |  | *Yes* | *No* | *No* |  |
| Primary cause of the bleed | | *Yes* | *Yes* | *Yes* | *Yes* | *Yes see comment* | *No* | *Yes* | *Did not include a primary cause so women had multiple causes* |
| *Blood products given* | |  |  |  |  |  |  |  |  |
| Number of units of RBC | | *Yes* | *Yes* | *Yes* | *Yes* | *Yes* | *Yes* | *Yes* |  |
| Fresh Frozen Plasma | | *Yes* | *Yes* | *Yes* | *Yes* | *Yes* | *Yes* | *Yes* |  |
| Platelets | | *Yes* | *Yes* | *Yes* | *Yes* | *Yes* | *Yes* | *Yes* |  |
| Cryoprecipitate | | *Yes* | *Yes* | *Yes* | *Not used* | *Not used* | *No* | *Yes* | *Included as interesting difference between countries* |
| Fibrinogen | | *Yes* | *Yes* | *Yes* | *Yes* | *Yes* | *No* | *Yes* |  |
| Cell Saver | | *Yes* | *Yes* | *No* | *No* | *No* | *No* | *Yes* | *Included as interesting difference between countries* |
| Factor VIIa | | *Yes* | *No* | *Yes* | *Yes* | *Yes* | *No* | *Yes* |  |
| Tranexamic acid | | *Yes* | *No* | *Yes* | *Yes* | *Yes* | *No* | *Yes* |  |
|  | |  |  |  |  |  |  |  |  |
| Colloid (ml) | | *Yes* | *No* | *Yes* | *Yes* | *Yes* | *No* | *Yes* |  |
| Crystalloid (ml) | | *Yes* | *No* | *Yes* | *Yes* | *Yes* | *No* | *Yes* |  |
| Time of first unit | | *No* | *Yes* | *No* | *Yes* | *No* | *No* | *No* |  |
| Time of eighth unit | | *No* | *Yes* | *No* | *Yes* | *No* | *No* | *No* |  |
| *Medical Management* | |  |  |  |  |  |  |  |  |
| Oxytocin | | *Yes* | *Yes* | *Yes* | *Yes* | *Yes* | *No* | *Yes* | *Italian data limited to vaginal births* |
| Ergometerine | | *Yes* | *Yes* | *No* | *Yes* | *No* | *No* | *Yes* |  |
| Prostaglandin | | *Yes* | *Yes* | *Yes* | *Yes* | *Yes* | *No* | *Yes* |  |
| Misoprostol | | *Yes* | *Yes* | *No* | *Yes* | *Yes* | *No* | *Yes* |  |
| *Management surgical* | |  |  |  |  |  |  |  |  |
| Suturing | | *Yes* | *No* | *No* | *No* | *No* | *No* | *No* |  |
| Placenta removal | | *Yes* | *No* | *No* | *No* | *Yes* | *No* | *No* |  |
| Intra-abdominal packing | | *Yes* | *Yes* | *No* | *Yes (not comparable)* | *Yes* | *No* | *Yes* |  |
| Balloons | | *Yes* | *Yes* | *Yes* | *Yes* | *Yes* | *Yes* | *Yes* |  |
| Embolisation and ligation | | *Yes* | *Yes* | *Yes see comment* | *Yes* | *Yes* | *Yes* | *Yes* | *Embolisation and ligation combined due to the UK data* |
| Intra-arterial balloons | | *Yes* | *Yes* | *No* | *No* | *No* | *Yes* | *No* |  |
| Uterine compression sutures | | *Yes* | *Yes* | *Yes* | *Yes* | *Yes* | *Yes* | *Yes* |  |
| Hysterectomy | | *Yes* | *Yes* | *Yes* | *Yes* | *Yes* | *Yes* | *Yes* |  |
| *Maternal morbidity* | |  |  |  |  |  |  |  |  |
| Death | | *Yes* | *Yes* | *Yes* | *Yes* | *Yes* | *Yes* | *Yes* |  |
| Serious maternal morbidity | | *Yes* | *Yes* | *Yes* | *No* | *See comment* | *No* | *No* | *Excluded outcomes were as a result of a lack of validity and case ascertainment* |
| Respiratory distress syndrome | | *Yes* | *Yes* | *Yes* | *No* | *Yes* | *No* | *No* |  |
| Coagulopathy | | *Yes* | *Yes* | *Yes* | *No* | *Yes* | *No* | *No* |  |
| Kidney failure | | *Yes* | *Yes* | *Yes* | *No* | *Yes* | *Yes* | *Yes* |  |
| Cardiac arrest | | *Yes* | *Yes* | *Yes* | *No* | *Yes* | *Yes* | *Yes* |  |
| Pulmonary oedema | | *No* | *Yes* | *Yes* | *No* | *Yes* | *No* | *No* |  |
| Thrombotic event | | *Yes* | *Yes* | *Yes* | *No* | *Yes* | *Yes* | *Yes* |  |
| Multiple organ failure | | *Yes* | *Yes* | *Yes* | *No* | *See comment* | *No* | *No* | *French data not comparable* |
| Septicaemia | | *Yes* | *Yes* | *Yes* | *No* | *Yes* | *No* | *Yes* |  |
| Required ventilation | | *No* | *Yes* | *Yes* | *No* | *Yes* | *No* | *No* |  |
| Pelvic injury | | *No* | *Yes* | *No* | *No* | *Yes* | *No* | *No* | *Not relevant or valid outcomes* |
| Reaction to transfusion | | *No* | *Yes* | *No* | *No* | *Yes* | *No* | *No* |  |
| Other morbidity | | *No* | *Yes* | *No* | *No* | *No* | *No* | *No* |  |
| ITU admission | | *Yes* | *Yes* | *Yes* | *Yes* | *Yes* | *No* | *Yes* |  |

S2 Table. Aetiology of the massive obstetric haemorrhage by country

|  | | **UK n=162** | | **AMOSS n=62** | | **Italy n=99** | | **the Netherlands n=179** | | **France n=126** | |
| --- | --- | --- | --- | --- | --- | --- | --- | --- | --- | --- | --- |
| Atony | | 64 | (39.5) | 25 | (40.3) | 56 | (56.6) | 112 | (62.6) | 46 | (36.5) |
| Placenta Praevia | | 12 | (7.4) | 4 | (6.5) | 0 | (0) | 4 | (2.2) | 3 | (2.4) |
| Abnormally invasive placenta | | 27 | (16.7) | 12 | (19.4) | 20 | (20.2) | 17 | (9.5) | 11 | (8.7) |
| Placenta abruption | | 15 | (9.3) | 4 | (6.5) | 3 | (3) | 5 | (2.8) | 10 | (7.9) |
| Uterine rupture | | 5 | (3.1) | 2 | (3.2) | 3 | (3) | 0 | (0) | 3 | (2.4) |
| Retained placenta | | 1 | (0.6) | 4 | (6.5) | 2 | (2) | 18 | (10.1) | 12 | (9.5) |
| Laceration | | 22 | (13.6) | 7 | (11.3) | 6 | (6.1) | 21 | (11.7) | 19 | (15.1) |
| Other | | 15 | (9.3) | 3 | (4.8) | 8 | (8.1) | 2 | (1.1) | 11 | (8.7) |
| Missing | | 1 | (0.6) | 1 | (1.6) | 1 | (1) | 0 | (0) | 11 | (8.7) |
|  |  |  |  |  |  |  |  |  |  |  |  |
| Atony | | 64 | (39.5) | 25 | (40.3) | 56 | (56.6) | 112 | (62.6) | 52 | (41.3) |
| Abnormal placentation | | 40 | (24.7) | 20 | (32.3) | 22 | (22.2) | 39 | (21.8) | 31 | (24.6) |
| Trauma | | 27 | (16.7) | 9 | (14.5) | 9 | (9.1) | 21 | (11.7) | 20 | (15.9) |
| Abruption | | 15 | (9.3) | 4 | (6.5) | 3 | (3) | 5 | (2.8) | 8 | (6.3) |
| Other | | 15 | (9.3) | 3 | (4.8) | 8 | (8.1) | 2 | (1.1) | 4 | (3.2) |
| Missing | | 1 | (0.6) | 1 | (1.6) | 1 | (1) | 0 | (0) | 11 | (8.7) |

France had a hierarchy of primary cause of PPH and were coded using this system 1st Abnormal placentation 2nd Abruption 3rd Trauma 4^th^ was Atony. Denmark did not have data on cause of haemorrhage.
