# Supplementary material for: A total blood volume or more transfused during pregnancy or after childbirth: Individual patient data from six international population-based observational studies
Source: PLoS One. 2021 Jan 22;16(1):e0244933. doi: 10.1371/journal.pone.0244933 (PMC7822517; doi:10.1371/journal.pone.0244933)
Supplement: S2 Table — Legend: France had a hierarchy of primary cause of PPH and were coded using this system: 1st Abnormal placentation, 2nd Abruption, 3rd Trauma and 4th was Atony. Denmark did not have data on cause of haemorrhage. (DOCX) [file pone.0244933.s002.docx]

S2 Table. Aetiology of the massive obstetric haemorrhage by country

|  | | **UK n=162** | | **AMOSS n=62** | | **Italy n=99** | | **the Netherlands n=179** | | **France n=126** | |
| --- | --- | --- | --- | --- | --- | --- | --- | --- | --- | --- | --- |
| Atony | | 64 | (39.5) | 25 | (40.3) | 56 | (56.6) | 112 | (62.6) | 46 | (36.5) |
| Placenta Praevia | | 12 | (7.4) | 4 | (6.5) | 0 | (0) | 4 | (2.2) | 3 | (2.4) |
| Abnormally invasive placenta | | 27 | (16.7) | 12 | (19.4) | 20 | (20.2) | 17 | (9.5) | 11 | (8.7) |
| Placenta abruption | | 15 | (9.3) | 4 | (6.5) | 3 | (3) | 5 | (2.8) | 10 | (7.9) |
| Uterine rupture | | 5 | (3.1) | 2 | (3.2) | 3 | (3) | 0 | (0) | 3 | (2.4) |
| Retained placenta | | 0 | (0) | 4 | (6.5) | 2 | (2) | 18 | (10.1) | 12 | (9.5) |
| Laceration | | 22 | (13.6) | 7 | (11.3) | 6 | (6.1) | 21 | (11.7) | 19 | (15.1) |
| Other | | 16 | (9.9) | 3 | (4.8) | 8 | (8.1) | 2 | (1.1) | 11 | (8.7) |
| Missing | | 1 | (0.6) | 1 | (1.6) | 1 | (1) | 0 | (0) | 11 | (8.7) |
|  |  |  |  |  |  |  |  |  |  |  |  |
| Atony | | 64 | (39.5) | 25 | (40.3) | 56 | (56.6) | 112 | (62.6) | 52 | (41.3) |
| Abnormal placentation | | 39 | (24.1) | 20 | (32.3) | 22 | (22.2) | 39 | (21.8) | 31 | (24.6) |
| Trauma | | 27 | (16.7) | 9 | (14.5) | 9 | (9.1) | 21 | (11.7) | 20 | (15.9) |
| Abruption | | 15 | (9.3) | 4 | (6.5) | 3 | (3) | 5 | (2.8) | 8 | (6.3) |
| Other | | 16 | (9.9) | 3 | (4.8) | 8 | (8.1) | 2 | (1.1) | 4 | (3.2) |
| Missing | | 1 | (0.6) | 1 | (1.6) | 1 | (1) | 0 | (0) | 11 | (8.7) |

France had a hierarchy of primary cause of PPH and were coded using this system 1st Abnormal placentation 2nd Abruption 3rd Trauma 4^th^ was Atony. Denmark did not have data on cause of haemorrhage.
